# Promoting cultural change towards dementia friendly communities: a multi-level intervention in Japan

**DOI:** 10.1186/s12877-022-03030-6

**Published:** 2022-04-23

**Authors:** Shuji Tsuda, Hiroki Inagaki, Tsuyoshi Okamura, Mika Sugiyama, Madoka Ogawa, Fumiko Miyamae, Ayako Edahiro, Chiaki Ura, Naoko Sakuma, Shuichi Awata

**Affiliations:** Research Team for Promoting Independence and Mental Health, Tokyo Metropolitan Institute of Gerontology, 35-2 Sakae-Cho, Itabashi-ku, Tokyo, 173-0015 Japan

**Keywords:** Dementia-friendly community, Dementia, Social capital, Social networks, Japan

## Abstract

**Background:**

Effective strategies to develop dementia-friendly communities (DFCs) are needed in aging societies. We aimed to propose a strategy to develop DFCs from a Japanese perspective and to evaluate an intervention program that adopted the strategy.

**Methods:**

This study implemented a multi-level intervention that emphasized nurturing community social capital in a large apartment complex in the Tokyo metropolitan area in 2017. We offered an inclusive café that was open for extended hours as a place to socialize and a center for activities that included monthly public lectures. Individual consultation on daily life issues was also available for free at the café. Postal surveys were sent out to all older residents aged 70 years and older in 2016 and 2019. With a one-group pre-test and post-test design, we assessed changes in the proportion of older residents who had social interaction with friends and those who were confident about living in the community, even if they were living with dementia.

**Results:**

Totals of 2633 and 2696 residents completed the pre and post-intervention surveys, respectively. The mean age of the pre-intervention respondents was 77.4 years; 45.7% lived alone and 7.7% reported living with impaired cognitive function. The proportion of men who had regular social interaction and were confident about living in their community with dementia increased significantly from 38.8 to 44.5% (*p =* 0.0080) and from 34.1 to 38.3% (*p =* 0.045), respectively. Similar significant increases were observed in the subgroup of men living with impaired cognitive function, but not in the same subgroup for women.

**Conclusions:**

The intervention benefitted male residents who were less likely to be involved in the community’s web of social networks at baseline. A strategy to create DFCs that emphasizes nurturing community social capital can form a foundation for DFCs.

**Trial registration:**

This study was retrospectively registered in the University hospital Medical Information Network (UMIN) Clinical Trial Registry (registry number: UMIN000038193, date of registration: Oct 3, 2019).

## Background

### The concept of dementia-friendly communities (DFCs) and current evidence

Over the past decades, the concept of ‘dementia friendliness’ has gained prominence. Recognizing that the obligations, inequalities, and vulnerabilities experienced by people living with dementia can lead to disability, the concept adopts a rights-based approach that is in line with the United Nations Convention on the Rights of Persons with Disabilities [1, 2]. Dementia friendliness values the human rights of people living with dementia and empowers them to participate in society. DFCs, an embodied form of the concept, refer to aggregated actions taken at various levels in society to reduce social stigma around dementia and to promote the social engagement of people living with dementia [1, 3]. DFCs are usually pursued at a community level (i.e. geographic locations). Research evidence shows that the effective size of a community to seek a cultural change towards DFCs is relatively small—an area with a population of no more than about 10,000 people over a distance of no greater than 10 km [3]. General recommendations on activities needed to achieve DFCs explain that it is essential to address multiple aspects of the target community including people, place, networks, and resources [3]. Since every community has its own unique features, a one-size-fits-all solution does not exist [4]. Actions towards DFCs could be more effective when they are tailored according to the needs of the target population, their cultural preferences, and existing community resources [5].

### Current situation around dementia and DFCs in Japan

Along with Japan’s aging population, the number of people living with dementia in 2012 was estimated at around 4.6 million and is projected to increase to 7 million by 2025; this means that one in five adults aged 65 and older will be living with dementia [6]. Anticipated issues associated with this upward trend were recognized early by the Japanese government and a series of social policies on dementia care have been implemented. In 2005, the government launched a nationwide campaign that focused on raising public awareness and offered an educational program to train various groups and individuals on how to best support people living with dementia and their caregivers in the community [7]. This awareness-raising program has been incorporated in subsequent dementia care policies and, as of September 2020, 12.7 million people have completed the certificate program [8]. Recent social policies on dementia care in Japan, ‘the Orange Plan’ and ‘the New Orange Plan’, have identified this awareness-raising program as one of the drivers that helps to create DFCs at a community level and places greater emphasis on bolstering regional actions to realize DFCs in each municipality [9, 10]. The two dementia policies have underlined the paramount importance of prioritizing the perspectives of people living with dementia and their families and requested their participation in the governmental meetings on dementia. The national government also enables local governments and private sectors to take collaborative actions towards DFCs, it has provided them with financial incentives, through which, grassroots activities such as dementia cafés have burgeoned across the country [11].

### A potential pathway to DFCs through enhanced social capital

Several DFCs with different approaches have been introduced and key features for successful DFCs have been reported in recent years [12–14]. These pieces of evidence suggest that community social capital could be a foundation for successful DFCs in which people living with dementia actively engage. A qualitative study revealed public views on what made a community in Ontario, Canada dementia-friendly [15]. This study considered the strong social networks available to people living with dementia, and the informal social support exchanged via these networks as a valuable asset to DFCs. Another research analyzed cross-sectional data from Japan and found that a higher degree of neighborhood ties and perceived social support in residential districts was associated with higher proportions of people with cognitive decline living in such districts [16]. This indicates that people living with dementia may feel more secure and more able to maintain their daily activities in communities, in which an abundant supply of social support is exchanged through neighborhood ties.

When referring to empirical evidence from experimental studies leveraging social capital for general older adults, a variety of interventions to improve their health outcomes have been proposed and tested [17]. These interventions were found to be effective in yielding healthy behaviors, such as the use of health-related resources and positive impacts on physical and psychological wellbeing in targeted older adults at the individual level [17]. Since the strategies, participants, and settings varied across the studies reviewed, there is a broad possibility relating to the application of this approach to older adults. Additionally, the potential advantage of taking this approach to a community-level intervention such as DFC creation would have a spillover effect, indicating that the effects of social capital could be delivered not only to the study participants, but also to those who, despite not being involved in the intervention, had some connections with the participants [18]. Social contagion—one of the theoretical pathways linking social capital to population health at the community level—explains that through a social network, perceptions and behaviors get diffused. Information and behavioral norms are disseminated through a spider net of social networks in a community and, thus, the members can adopt new behaviors. The Framingham offspring study proved this pathway by demonstrating that smoking cessation behaviors disseminated through social networks had ‘three degrees of influence’, meaning a behavioral change translated to an index person’s friends’ friends’ friends [19].

DFC activities aim to produce cultural and structural changes in a community. This could be accomplished by applying social capital interventions for connecting members across the community, including people with dementia, and radiating positive views on dementia and healthy behaviors such as giving mutual support through the web of networks.

### Social capital from a Japanese perspective

Community social capital has been a key ingredient that has a salient impact on people and society in Japan. The quick recovery from the massive earthquake was made possible by local neighborhood associations in the affected communities [20]. Social capital shared among the members of a residential area has a preventive effect against committing suicides [21, 22]. In community-dwelling Japanese older adults, participating in community salons boosts their self-rated health and prevents them from functional decline [23, 24].

Typical structures that have nurtured community social capital in Japan used to be neighborhood-related civic activities such as community festivals, fire-prevention patrols in winter, and garbage collection management [25]. Through such activities, the Japanese tend to create small, cohesive groups, which can provide reassurance to live in the neighborhood and exchange mutual support. However, such civic activities’ frequency and the time spent on them became shorter due to the lifestyle changes caused by the rapid economic growth during the late twentieth century [25]. A lack of facilitators of these conventional activities due to an aging population combined with the diminishing number of children, amplified this trend in the twenty-first century, and drove the withdrawal of these activities in many communities [26].

Alternative structures, such as neighborhood volunteering groups, comprehensive sports clubs, and community salons have emerged [27]. These alternatives tend to take root in small residential areas from which members are convened, just like the neighborhood-related civic activities did so in the past [26]. Presumably, the benefits from engagement in these alternative activities would be similar to that from the outdated ones, as some scientific evidence exists on higher rates of social participation and better psychological well-being found in those participating in the contemporary ones [23, 28, 29]. From this historical perspective, lively neighborhood activities shape a reassuring and inclusive community, which we assumed can lay the groundwork for DFCs in Japan.

### Needing effective models for promoting cultural changes towards DFCs

Thus far, much of the evidence on DFCs have come from conceptual discussions and findings from observational studies [4, 30]. These have clarified the characteristics of DFCs and the facilitators and barriers to community engagement for people living with dementia. A few experimental studies on DFCs evaluated the effects of DFC activities on the study participants who were directly involved [14, 31]. Systematic approaches to outcome evaluations are still lacking. More specifically, scarce evidence exists on what type of community-wide DFC interventions yield a positive impact on the population at large, at a community level.

### Aim of this study

This study examined from an ecological perspective the community-level effect of a multi-level DFC intervention with a particular emphasis on developing the community social capital of all the older adults living in the targeted community.

### Research hypotheses

1. We hypothesized that effective DFC activities would produce social interactions across diverse individuals in the targeted community and expand the web of diverse social networks, which embrace people living with dementia.
2. Activated interaction among community members would promote a collective sense of confidence in living in their community despite living with disabilities due to dementia.
3. The same interaction would raise dementia awareness in the community and spread knowledge about community resources for people with dementia.

## Methods

### Study design

We conducted a multi-level DFC intervention in close collaboration with the local government and healthcare professionals (HCPs) in the community. The intervention was developed and tailored according to the characteristics and needs of the community, with particular attention to enhancing community social capital. The effects of the intervention were evaluated with a one-group pre-test and post-test design using repeated cross-sectional data collected from the entire older population in the community. To assess community-level impacts of the intervention, we conducted ecological analyses that used aggregated measures summarizing the observations derived from both participants and non-participants.

### Setting and population

This study was conducted in a large apartment complex located in the Tokyo metropolitan area. This complex was selected as an intervention site owing to its size—population and area—comprising a suitable unit to hold DFC activities, and since its weathered systems for nurturing the social capital needed to be updated to satisfy the current residents’ needs. The complex was built in the early 1970s and included 8000 rental family housing units in 28 apartment buildings within a 3000 m^2^ block. When it was first built, the complex attracted many young families and the population peaked at 18,000 in the early 1990s. The population declined as the building aged and is currently estimated at 13,000 with 43% of the residents aged 65 and older. Residents in the complex used to hold a variety of neighborhood-related civic activities, through which they built dense networks that supposedly contributed to creating and reinforcing social capital. However, the population’s aging and erosion have caused these activities to either shrink or be abandoned.

We sent out the first survey in 2016 to assess the characteristics and needs of the older residents living in the complex. The survey revealed that the complex had a high proportion of older adults living alone and people with a low household income, as shown in Table 1. The assessment survey also showed low frequencies of social interaction or low degrees of perceived social support among older residents in the complex. From the survey, we concluded that older residents in this community needed a scheme to increase social interaction between the members and to broaden the web of social networks in the community. Detailed information on how we assessed community needs and designed our intervention is described elsewhere [32].

**Table 1:** Basic characteristics of the entire respondents in the 2016 and 2019 surveys

	Male					Female				
	Pre_2016 (*n* = 1091)		Post_2019 (*n* = 1091)			Pre_2016 (*n* = 1542)		Post_2019 (*n* = 1605)		
	n/mean	%/SD	n/mean	%/SD	p^a^	n/mean	%/SD	n/mean	%/SD	p^a^
Age in years	76.7	(4.8)	77.0	(5.2)	0.20	77.9	(5.5)	77.8	(5.6)	0.38
Married	694	(64.7%)	636	(61.3%)	0.11	553	(36.5%)	557	(36.7%)	0.91
Living alone	380	(35.3%)	410	(38.5%)	0.14	822	(53.8%)	842	(54.7%)	0.66
Years of residence	28.6	(15.7)	28.7	(17.1)	0.86	26.6	(15.5)	28.8	(16.4)	< 0.001
Being employed	321	(30.1%)	332	(31.1%)	0.64	184	(12.2%)	257	(16.4%)	< 0.001
Household income ≥3 million yen	233	(24.3%)	241	(23.7%)	0.79	170	(13.6%)	190	(13.8%)	0.91
Intact cognitive function	996	(91.9%)	947	(88.3%)	0.0060	1433	(93.7%)	1395	(87.9%)	< 0.001
Not depressed (5-item GDS < 2)	569	(56.4%)	548	(56.1%)	0.89	810	(57.7%)	869	(58.8%)	0.57
Number of comorbidities, none	109	(10.1%)	72	(6.7%)	0.011	174	(11.3%)	142	(9.0%)	0.12
1 comorbidity	271	(25.0%)	242	(22.6%)		396	(25.8%)	381	(24.3%)	
2 comorbidities	278	(25.6%)	271	(25.3%)		349	(22.7%)	390	(24.8%)	
3 comorbidities	189	(17.4%)	215	(20.1%)		255	(16.6%)	260	(16.5%)	
≥4 comorbidities	237	(21.9%)	271	(25.3%)		361	(23.5%)	398	(25.3%)	
Number of impaired IADL items, none	538	(49.3%)	463	(42.4%)	< 0.001	910	(59.0%)	942	(58.7%)	< 0.001
1 item impaired	305	(28.0%)	289	(26.5%)		361	(23.4%)	314	(19.6%)	
2 items impaired	241	(22.1%)	289	(20.1%)		260	(16.9%)	180	(11.2%)	
3-5 items impaired	7	(0.6%)	120	(11.0%)		11	(0.7%)	169	(10.5%)	

*SD* Standard deviation *GDS* Geriatric depression scale, *IADL* Instrumental activities of daily living

^a^Chi-square test or two sample t-test

### Aim of the intervention

The aim of our intervention was to rejuvenate the apartment complex to be a compassionate and inclusive community, where older residents including those with dementia could feel a sense of reassurance in living and participating. We intended to achieve this by creating a scheme to increase social interactions between residents with diverse backgrounds, expand the web of social networks in the community, and diffuse dementia-friendly views and attitudes. We anticipated that favorable views and attitudes permeate the entire community through newly developed social ties, as well as via existing ties from outside the intervention (i.e. social contagion and spillover effects).

### Intervention

We designed a multi-level intervention: four community-level components consisting of 1. an extended hour, freely accessible café that was welcoming to people living with dementia, 2. monthly open lectures and workshops about inclusive communities and healthy aging, 3. connecting community resources and professionals, 4. nurturing dementia supporters, and an individual-level component 5. offering free consultation sessions on daily life issues with HCPs at the café and outreach support for social issues was also provided individually when the HCPs felt it necessary. Of these five elements, the café played a central role in our intervention, as it was placed at the heart of the complex and served as a hub for all the activities.

#### Intervention 1

The café was located on the ground floor of the building facing the central square of the block and was open from 11 a.m. to 4 p.m. three or 4 days a week, which allowed both passers-by and regular users to drop in whenever they wanted to and to participate in activities held voluntarily by the users. Examples of the activities were improvisational chorus singing by a random group of participants and regularly scheduled activities such as Japanese chess and handcrafting. To help people with special needs including people living with dementia to engage in mainstream community life at the café, HCPs— social workers and clinical psychologists in our research team and from the community—worked in shifts to observe the activities and to assist those with special needs to make friends with other café users. We intended the café to serve as an ignition, through which residents with diverse backgrounds could develop new social ties under a safe environment, and from which the café users could activate neighborhood interactions and invite non-users into the web of social networks in the community.

#### Intervention 2

Public lectures and workshops on inclusive communities and healthy aging were provided monthly at the café. These offered some activities on the topic discussed and encouraged the participants to work together in groups. The intention behind this was to disseminate the notion of dementia friendliness and inclusive societies, and simultaneously, to establish new social ties.

#### Intervention 3

Community HCP meetings also took place at the café. This was meant to enhance their communication and collaboration, which could result in an efficient provision of formal support and provide reassurance of living in the community.

#### Intervention 4

We hosted monthly training sessions for formal and informal dementia supporters in the community. This component was designed to raise awareness and knowledge of the participants who could offer an additional layer of individual support and reassurance to people with dementia.

#### Intervention 5

The HCPs who were deployed at the café also gave informal individual consultation sessions, which were freely available to anyone who wanted to discuss their daily life issues. Medical and dental doctors also offered free verbal consultations, without prescribing or providing medical procedures, once a week. These sessions took place either at a corner of the café or in a separate, private room next to it.

The HCPs and doctors who provided the intervention including the consultation sessions had substantial years of clinical experience and they also received training sessions on how to support community-dwelling older adults who were living with dementia before the program was launched [33]. The topics discussed in these training sessions varied: physical and psychological problems, mistrust of medical services, questions on the systems of medical and long-term care services, problems regarding memory and cognitive function, and family and caregiving issues [34].

We opened the café in April 2017 and started all the components of our intervention simultaneously. All elements of the intervention were being served at the time of the evaluation survey. On average, 11.6 and 29.7 people visited the café every day in the first and second year. The total numbers of consultation sessions were 247 and 598 in each year and the mean numbers of participants in the monthly public lectures were 57.1 and 51.3 [33]. Of these users of our program, more than 80% came from the apartment complex under study, 90% were 70 years or older, and two-thirds were female.

### Dataset and measures

To evaluate the effects of the intervention, we adopted a one-group pre-test and post-test design with repeated cross-sectional data collected via postal mail. We first approached the ward office for the individual information of the entire older population living in the ward where the apartment complex was located and then sent out postal surveys in 2016 and 2019 to the entire population aged 70 or older. The analyses of this study only used extracted data from those who lived in the apartment complex. A considerable proportion of respondents in the two waves overlapped; however, due to logistic issues behind the study, we were unable to connect the individual data across waves or compose panel data.

#### Outcome measures

This study evaluated three sets of outcomes pre (2016) and post (2019) intervention: social interaction, confidence in living with dementia in the community, and awareness of dementia. We selected social interaction as a social capital measurement because it explains the pathway through which social contagion and spillover effects occur. From ecological perspectives, the proportions of those with direct and indirect social interactions indicate the degree of activeness with which community members interact. We assumed these aggregated measures were community-level indicators of what proportion of residents were actively involved in the web of social networks in the community. This was the immediate outcome of the intervention that would alter the other two outcomes (collective sense of confidence and dementia awareness in the community) by the mechanism of social contagion.

The surveys contained two questions asking about the frequency of social interaction with friends: direct in-person interaction and indirect interaction on the phone or via messaging. The following five response options were provided; ‘twice a week or more’, ‘once a week’, ‘a few times a month’, ‘once a month’, and ‘less than once a month’. For the analysis, the responses were dichotomized to ‘once a month or more’ or ‘less than once a month’.

To examine the collective sense of confidence in living in the community despite living with dementia, we asked the question: ‘How confident are you about living in your community even if you get dementia?’ with five Likert-like responses ranging from ‘not confident at all’ to ‘very confident’. The answers were dichotomized as ‘confident’ or ‘not confident’. We regarded the proportion of those with confidence as a community-level indicator of perceived dementia friendliness.

To investigate whether dementia awareness was raised in the community, we employed the following three questions: ‘Are you aware of what dementia symptoms are like?’, ‘Are you aware of how to communicate with people with dementia?’ and ‘Are you aware of whom you should consult with when you have trouble with dementia?’ Responses were given on a 4-point Likert scale from ‘not at all aware’ to ‘very aware’ with a score of 1 as the lowest level of awareness and 4 as the highest. Mean scores were computed to indicate the degree of dementia awareness in the community.

#### Measures of the basic characteristics

In the surveys in both waves, the following questions on basic characteristics were offered: age in years, sex, marital status, household composition, years of residence, employment status, and household income. We observed these individual measures so that the proportions and mean scores illustrate the socioeconomic status of the apartment complex. For current marital status, we asked respondents whether they had a spouse living with them, lived alone, or if they were living with a common-law partner. A question on household composition provided three response options: living alone, couple, or couple with other family members, and the responses were dichotomized as ‘living alone’ or ‘otherwise’. We offered a question on employment status with three response options: working 35 h or more a week, working less than 35 h a week, or not working, and the answers were dichotomized as ‘working’ or ‘not working’. For the household income question, seven response options were given from zero to more than 10 million yen per year. We dichotomized the answers with a cut-off point of 3 million yen.

#### Measures of physical, psychological, and cognitive status

Survey measures assessing respondents’ physical, psychological, and cognitive status included the number of comorbidities, instrumental activities of daily living (IADL), depressive symptoms, and cognitive function. The number of comorbidities was measured by adding up diseases and conditions from a list of 18 items. Impairment in IADL was assessed using the corresponding five questions from the Kihon Check List, which is a validated and widely applied self-rated questionnaire to evaluate frailty in community-dwelling older adults in Japan [35]. These five items asked about abilities of using public transportation, shopping for daily necessities, handling bank accounts, housekeeping, and making phone calls. Depressive symptoms were assessed using the Japanese version of the 5-item Geriatric Depression Scale (GDS). A validated cut-off score of 2 was adopted to interpret the GDS, meaning that having more than two depressive symptoms indicated a depressive tendency [36]. To assess cognitive function, we used a self-administered dementia checklist consisting of 10 items assessing whether respondents had problems with memory and instrumental activities of daily living [37]. The checklist offers a 4-point Likert-like scale for each item and returns scores from 10 to 40, with a higher score indicating worse cognitive function. We set a cut-off value of 17/18, which was found to be a significant discriminative threshold against the Clinical Dementia Rating scores in a validation study [38].

### Statistical analysis

The respondents’ characteristics in each wave were summarized descriptively and compared between pre and post to assess whether there were any changes in the population characteristics in the community. Cross-tabulation with chi-squared tests was used to compare binary outcomes pre and post-intervention. To analyze changes in continuous outcomes, two-sample t-tests were performed.

Since we were interested in the community-level effects of implementing the intervention, we selected ecological (community-level) analyses, whose unit of measurement was individuals but unit of analysis was groups [39]. From individual-level data aggregated from the surveys, we summarized the proportions and the mean scores to indicate community-level outcomes. With the summary data, we analyzed community-level changes between before and after the implementation of the intervention. We conducted main analyses of the entire older population and subgroup analyses of people experiencing cognitive decline according to the scores in the self-rated dementia checklist. All the analyses were stratified by sex, interpreted with the level of statistical significance set at *p <* 0.05, and computed using IBM SPSS Statistics v.23.

## Results

### Characteristics

A total of 2633 older residents completed the 2016 survey (response rate, 67.9%), compared with 2696 in the 2019 survey (response rate, 66.6%). Table 1 summarizes the basic characteristics of the respondents in the two surveys. The mean age of male respondents in 2016 and 2019 was 76.7 (standard deviation, SD 4.8) and 77.0 (SD 5.2), while for female respondents the mean age was 77.9 (SD 5.5) and 77.8 (SD 5.6), respectively. The proportion of residents living alone was lower among males, as 35.3 and 38.5% lived alone in each survey, while 53.8 and 54.7% of female residents lived in single households, respectively. For both sexes, about 10% showed impairment in cognitive function on the self-rated dementia checklist in both waves, and the prevalence of depressive tendencies was relatively high, as more than 40% of respondents in both waves reported two or more depressive symptoms out of the five GDS questions.

The subgroup characteristics of older respondents living with impaired cognitive function are summarized in Table 2. The mean ages of male and female respondents in the two surveys were between 79.5 and 83.4 years. About 70% of male and 35% of female respondents living with impaired cognitive function were married. The proportion of respondents who had a single household was 24.4% in 2016 and 29.3% in 2019 for men, and 35.8 and 52.2% for women. Approximately 80% of male and female respondents living with impaired cognitive function had more than two depressive symptoms.

**Table 2:** Subgroup characteristics of those with impaired cognitive function in the 2016 and 2019 surveys

	Male					Female				
	Pre_2016 (*n* = 88)		Post_2019 (*n* = 125)			Pre_2016 (*n* = 97)		Post_2019 (*n* = 192)		
	n/mean	%/SD	n/mean	%/SD	p^a^	n/mean	%/SD	n/mean	%/SD	p^a^
Age in years	79.5	(5.5)	80.0	(5.6)	0.52	83.4	(7.2)	82.7	(6.0)	0.34
Married	62	(71.3%)	77	(65.8%)	0.45	35	(37.2%)	58	(32.2%)	0.42
Living alone	21	(24.4%)	36	(29.8%)	0.43	34	(35.8%)	96	(52.2%)	0.011
Years of residence	25.8	(16.6)	29.3	(17.6)	0.16	22.7	(15.4)	25.1	(15.6)	0.23
Being employed	5	(5.8%)	15	(12.2%)	0.15	0	(0.0%)	3	(1.6%)	0.55
Household income ≥3 million yen	9	(13.4%)	20	(18.9%)	0.41	4	(5.5%)	14	(9.1%)	0.44
Not depressed (5-item GDS < 2)	14	(20.0%)	23	(21.9%)	0.85	12	(15.2%)	49	(28.7%)	0.026
Number of comorbidities, none	4	(4.5%)	2	(1.7%)	0.030	8	(8.3%)	5	(2.6%)	0.14
1 comorbidity	15	(17.0%)	16	(13.2%)		16	(16.7%)	37	(19.4%)	
2 comorbidities	14	(15.9%)	33	(27.3%)		25	(26.0%)	38	(19.9%)	
3 comorbidities	27	(30.7%)	20	(16.5%)		13	(13.5%)	30	(15.7%)	
≥4 comorbidities	28	(31.8%)	50	(41.3%)		34	(35.4%)	81	(42.4%)	
Number of impaired IADL items, none	4	(4.5%)	14	(11.2%)	< 0.001	7	(7.2%)	14	(7.3%)	< 0.001
1 item impaired	18	(20.5%)	13	(10.4%)		27	(27.8%)	19	(9.9%)	
2 items impaired	64	(72.7%)	30	(24.0%)		61	(62.9%)	38	(19.8%)	
3-5 items impaired	2	(2.3%)	68	(54.4%)		2	(2.1%)	121	(63.0%)	

*SD* Standard deviation, *GDS* Geriatric depression scale, *IADL* Instrumental activities of daily living

^a^Chi-square test or two sample t-test

### Social interaction

Table 3 shows the differences in outcomes between pre and post-intervention among all respondents, and Table 4 shows the results of subgroup analyses of those living with impaired cognitive function. Male respondents who engaged in face-to-face social interaction with friends once a month or more increased significantly from 38.8 to 44.5% in the overall population (*p =* 0.0080) and from 9.8 to 21.6% in the subgroup analysis (*p =* 0.033). These results suggest that the proportion of male respondents with and without dementia, who were involved in the web of social networks in the community increased significantly. However, this effect was not significant among female respondents (58.2 and 60.2%, *p =* 0.27), and was almost significant among female respondents living with impaired cognitive function (20.0 and 30.7%; *p =* 0.081). Engagement in indirect social interaction with friends through phones and messages increased slightly in both sexes in the overall and subgroup analyses, but these changes were not statistically significant.

**Table 3:** Changes in outcomes among the entire respondents

	Male					Female				
	Pre_2016 (*n* = 1091)		Post_2019 (*n* = 1091)			Pre_2016 (*n* = 1542)		Post_2019 (*n *= 1605)		
	n/mean	%/SD	n/mean	%/SD	p^a^	n/mean	%/SD	n/mean	%/SD	p^a^
Number of people who have in-person social interaction with friends ≥1/month	412	(38.8%)	468	(44.5%)	0.0080	860	(58.2%)	925	(60.2%)	0.27
Number of people who have indirect social interaction with friends ≥1/month	553	(52.1%)	586	(55.5%)	0.13	1048	(70.5%)	1095	(71.4%)	0.58
Number of people who are confident in living with dementia in the community	362	(34.1%)	393	(38.3%)	0.030	462	(30.9%)	508	(32.9%)	0.24
Awareness of dementia symptoms	2.96	(0.78)	2.99	(0.73)	0.35	3.03	(0.73)	3.04	(0.67)	0.88
Awareness of communication with people with dementia	2.26	(0.81)	2.22	(0.78)	0.33	2.44	(0.78)	2.42	(0.75)	0.32
Awareness of contact person to consult with on dementia	2.18	(0.85)	2.21	(0.83)	0.41	2.31	(0.85)	2.36	(0.83)	0.15

^a^Chi-square test for binary outcomes and two sample t-test for scores

*SD* Standard deviation

**Table 4:** Changes in outcomes among older adults with impaired cognitive function

	Male					Female				
	Pre_2016 (*n* = 88)		Post_2019 (*n* = 125)			Pre_2016 (*n* = 97)		Post_2019 (*n* = 192)		
	n/mean	%/SD	n/mean	%/SD	p^a^	n/mean	%/SD	n/mean	%/SD	p^a^
Number of people who have in-person social interaction with friends ≥1/month	8	(9.8%)	25	(21.6%)	0.033	18	(20.0%)	55	(30.7%)	0.081
Number of people who have indirect social interaction with friends ≥1/month	15	(18.5%)	32	(27.1%)	0.18	30	(33.7%)	78	(43.1%)	0.15
Number of people who are confident in living with dementia in the community	19	(23.5%)	45	(39.1%)	0.045	25	(27.8%)	58	(31.7%)	0.58
Awareness of dementia symptoms	2.61	(0.94)	2.61	(0.77)	0.98	2.77	(0.87)	2.66	(0.77)	0.27
Awareness of communication with people with dementia	2.00	(0.86)	1.98	(0.74)	0.88	2.21	(0.80)	2.02	(0.74)	0.056
Awareness of contact person to consult with on dementia	1.80	(0.88)	1.90	(0.85)	0.43	2.11	(0.84)	1.95	(0.79)	0.11

*SD* Standard deviation

^a^Chi-square test for binary outcomes and two sample t-test for scores

### Confidence in living with dementia in the community

Consistent with the results of face-to-face social interaction, male respondents in 2019 were significantly more likely to report their confidence in living with dementia in their community than those in 2016 in the overall population (34.1 and 38.3%; *p =* 0.045) and the subgroup analysis (23.5 and 39.1%; *p =* 0.030) (Tables 3 and 4). For female respondents in both analyses, we found non-significant increases in the proportion of those who shared this confidence. These results indicate that positive views on dementia friendliness had been disseminated among the male respondents.

### Dementia awareness

We evaluated three awareness scores regarding dementia symptoms, communication with people living with dementia, or contact persons to consult on dementia. There were no significant changes in these scores of awareness among respondents of both sexes in the overall population (Tables 3 and 4). These scores tended to decrease post-intervention among female respondents with impaired cognitive function, although this trend was not statistically significant.

## Discussion

In this study aiming at creating a DFC, we selected an apartment complex in the Tokyo metropolitan area with an aging population and degrading social capital as a targeted community, and implemented a multi-level intervention weighted towards reactivating community social capital. We evaluated whether the effect of the intervention covered all the older residents in the complex. The intervention was significantly effective in boosting social interaction and confidence in living with dementia in the community among male residents with or without impaired cognitive function. No such effects were observed among female residents, regardless of their cognitive function. The intervention did not increase awareness of dementia in the community.

### Differential effects between sexes on in-person social interaction

A significant increase in in-person social interaction with friends was observed only among male residents. The potential reason behind this differential effect between sexes is that social interaction at baseline was already high among women and this resulted in a non-significant increase. This interpretation may be justified by the subgroup analysis of female residents living with impaired cognitive function. Their baseline level of social interaction was only 20%, which was much lower than that of all female residents (58%), and their increase in social interaction was almost significant (*p =* 0.081). Seen from the angle of community-level changes, the web of social networks shared by the complex’s residents absorbed new members and the size of the web grew. The intervention also succeeded in encompassing people living with dementia in the web of networks and making it inclusive.

The differential effect between sexes is consistent with findings from an earlier study that proved the effectiveness of community-level interventions on social activities of community-dwelling older adults in Japan [28]. Although the purpose of their study was not to create a DFC, they evaluated the effects of municipality-level interventions aimed at producing social activities and found that only male residents significantly increased their frequency of social interaction. Their data analysis also found that the already high social interaction at baseline among women could explain the non-significant increase in social interaction among women.

### Social interaction: a potential intermediate factor to perception on dementia friendliness

Concurrently with the differential effect in social interaction, only male residents who had increased social interaction with friends gained confidence in living with dementia in their community. This suggests that the members’ perceived dementia friendliness grew with an expanded web of neighborhood ties. The theoretical pathway of social contagion explains how the perceptions and behaviours were disseminated through the web of social networks. A preferable perception of dementia friendliness can spread through an inclusive web that embraces people living with dementia. We must admit that this pathway warrants a more rigorous analysis because our repeated cross-sectional data, where individuals were not connected across waves, did not allow for a detailed analysis. However, this pathway is plausible when referring to the coherent narratives from people living with dementia in prior qualitative studies, who expressed that the vital function of a dementia-friendly neighborhood was connecting them with peers and neighbors, and facilitating meaningful interactions [5]. DFCs will be achievable when inclusive networks promote compassionate interactions and mutual exchange of support, thereby giving a sense of assurance of living in the community [15, 40].

### No changes in dementia awareness in the community

Unlike other outcomes, there was no substantial change in the three dementia awareness scores in this study. There can be two interpretations: either the dose of education given in the intervention was sub-optimal, or the ceiling effects of a pre-existing governmental awareness campaign in Japan prevented a further increase in the awareness scores. Referring to a relevant study conducted in Kiama, Australia, the former interpretation is more plausible. The Kiama study tested a community-level multi-component DFC intervention that put a great deal of effort into awareness campaigns and education in community organizations [41]. They found that random samples from the community did not report a significant increase in dementia awareness scores pre and post-intervention, while attendees of education sessions earned significantly greater scores than the random samples post-intervention. Even with a greater dose of awareness-raising and education components in community-based interventions, a statistically significant increase in awareness would be difficult to achieve with an ecological analysis.

### Strength and limitations

What is novel about this study is that an ecological analysis with survey data derived from the entire older population in the targeted community captured community-level effects of a DFC intervention. In countries with an aging society, many attempts have been made to pursue DFCs, but few community-level analyses have been conducted to assess their efforts [31]. With the ecological analysis of a large sample data, our study provides valuable insights on how to leverage social capital for DFC activities.

The study limitations need to be addressed. Firstly, the evaluation was conducted with the one-group pre-test and post-test study design that poses limitations in causal inference. This design was chosen because no comparable data from communities with similar characteristics were available. Even though we conducted the same surveys in the neighborhood communities surrounding the apartment complex, the basic characteristics such as socioeconomic status were critically different from each other and we considered it inappropriate to make it as a comparison. The study’s design without control groups was susceptible to potential threats of internal validity, including history threats and secular trends. The results could be influenced by external events, such as a launch of other social services for older adults, that occurred during the course of this study (i.e. history threats). However, the researchers and HCPs from the community were not aware of such influential events within the apartment complex or surrounding communities during the study period. Underlying trends of social awareness on dementia due to the New Orange Plan, the governmental dementia policy to encourage a social change and support the lives of people living with dementia in Japan, was inevitable (i.e. secular trends) [10]; hence, we cannot differentiate the effect of the policy from that of our intervention.

Secondly, the repeated cross-sectional data did not allow for individual-level analyses. It was impossible to estimate the effect of the intervention on each individual or to weigh the effect of intervention by exposure levels, such as frequency of visiting the café. However, the large sample size made it possible to compute the subgroup analyses, which led to a solid conclusion.

Thirdly, the sample attrition was relatively high (around 70% responded to each survey). This could result in higher proportions and scores in outcomes than the true numbers, since reasons for non-response may be indifference to the study outcomes (social interaction, dementia friendliness, and dementia awareness). However, when considering its effect on the comparisons between pre- and post-intervention, the effect should be nondifferential. This is because response rates were almost identical between waves, and the participants’ characteristics in each survey seemed to be analogous, in accordance with Table 1, which exhibits similar socio-economic status while most of the differences in characteristics were attributable to aging (e.g., years of residence, cognitive function, and the number of comorbidities).

Another limitation is the choice of the outcome measurements. The outcomes were not measured with standardized scales. The awareness scores, for example, might not have been sensitive enough to detect small changes, and could have been one of the reasons behind the non-significant changes. Additionally, the study investigated only one aspect of social capital, (the degree of social interaction). We chose this outcome because social contagion and spillover effects occur through social interaction. Social capital is conceptualized from different axes such as cognitive and structural social capital, or bonding, linking, and bridging social capital [18]. Assessment of social capital from different aspects (e.g., exact social network size or quality of social ties) would have allowed more rigorous analysis.

We are currently conducting another wave of survey from the same cohort to evaluate individual-level effects of the intervention program. The ongoing survey will address these issues described above by drawing data, including frequencies of involvement in the intervention program and outcome measures such as individual-level cognitive social capital.

### Implications

DFC activities require some modifications in the perceptions and behaviors of community members. A web of inclusive social networks, in which patients living with dementia actively engage can be a channel through which perceived stigma around dementia is eliminated and more favorable views on it are disseminated across the community. One potential strategy to expand such networks is providing various activities at an open, inclusive café under some observation and individualized support from HCPs.

## Conclusions

A multi-level DFC intervention weighted towards building a structure to nurture community social capital was effective in activating social interaction among male residents in the community. The intervention resulted in disseminating favorable perceptions of dementia friendliness among them. This approach particularly benefitted those who were socially inactive at the baseline. Intervention programs leveraging community social capital have the potential of solidifying a foundation for successful DFCs. Future experimental studies employing rigorous evaluation methods would provide conclusive evidence for this approach.

## Data Availability

The datasets used and analyzed during current study are available from from the corresponding author on reasonable request.

## Abbreviations

DFC: Dementia friendly community
HCP: Health care professional
IADL: Instrumental activities of daily living
GDS: Geriatric depression scale
SD: Standard deviation
